# Pattern electroretinogram, blue-yellow visual evoked potentials and the risk of developing visual field defects in glaucoma suspects: a longitudinal “survival” analysis with a very long follow-up

**DOI:** 10.1007/s00417-023-06364-y

**Published:** 2024-01-06

**Authors:** Cord Huchzermeyer, Robert Lämmer, Christian Y. Mardin, Friedrich E. Kruse, Jan Kremers, Folkert K. Horn

**Affiliations:** 1 Department of Ophthalmology, Universitätsklinikum Erlangen, Erlangen, Bayern Germany; 2 Medical Faculty, Friedrich-Alexander-Universität Erlangen-Nürnberg, Erlangen, Bayern Germany

**Keywords:** Glaucoma, Electroretinogram, Visually evoked potentials, Perimetry, Survival analysis

## Abstract

**Purpose:**

Estimating glaucoma suspects’ risk for visual field defects helps to avoid under- and over-treatment. In this retrospective, longitudinal cohort study with a very long follow-up, we studied whether pattern electroretinograms (PERG) amplitudes and blue-on-yellow visual evoked potential (BY-VEP) latencies can predict visual field defects.

**Methods:**

Participants of the Erlangen Glaucoma Study were examined with PERG and BY-VEP between 9/1991 and 8/2001. Stimuli were created using an optical bench with Maxwellian view and consisted of vertical gratings (0,88 cpd) in a 32° field for both PERG and BY-VEP. Patients were treated according to clinical standards and performed standard automated perimetry (SAP) annually. Retrospectively, patients with normal SAP at baseline were selected. Primary endpoint was conversion to perimetric glaucoma. Predictive value was modeled using Kaplan–Meier analyses and a multivariate cox proportional hazards model with the continuous variables PERG amplitude, BY-VEP peak time and SAP square-root of loss variance (sLV) after stratification for Jonas classification of the optic discs.

**Results:**

Of 412 patients (288: Jonas 0, 103: I, and 21: II; baseline age: 20–60 years), 65 converted to perimetric glaucoma during follow-up (0.5–23.3 years; median 5.5 years). Optic disc classification was a strong risk factor for conversion (log rank *p* < 0.0001), and patients with more advanced changes progressed earlier. In the multivariate analysis (log rank *p* = 0.005), only PERG amplitude remained an independent risk factor after stratification for optic disc morphology (*p* = 0.021), with a ~ 30% higher risk per μV amplitude decrease.

**Conclusions:**

PERG helps to estimate glaucoma suspects’ risk for visual field defects.

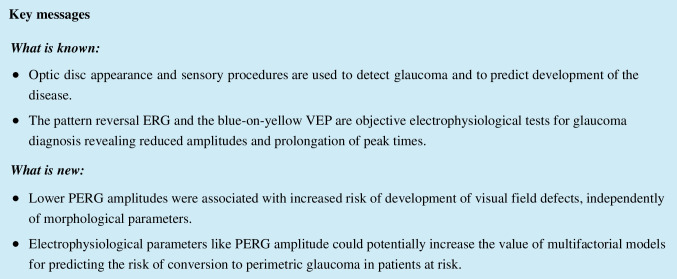



## Introduction

Glaucoma is heterogeneous group of progressive optic neuropathies related to intraocular pressure with characteristic features like cupping of the optic nerve head [1]. It is one of the most frequent causes of blindness worldwide [2]. Early treatment is warranted, because progression to blindness may occur at late stages even under therapy.

However, there is no simple biomarker for glaucoma. Instead, glaucoma is a clinical diagnosis which is based on the demonstration of distinctive structural and functional changes [1], and the diagnosis is often uncertain at early stages when only some but not all features are present [3], potentially leading to under- or overtreatment [4].

White-on-white standard automated perimetry is the gold standard for diagnosing functional damage in glaucoma. Its main limitation is the considerable intra-individual variability. Therefore, functional changes often cannot be demonstrated reproducibly and with adequate specificity before marked morphological changes are seen. Glaucoma without definite visual field defects is called pre-perimetric glaucoma. Still, on average, a cohort of patients with preperimetric glaucoma often shows significantly deterioration in functional parameters compared to a group of normal subjects, even if visual fields are not graded abnormal according to a given set of criteria. For example, these visual fields are often more variable, which is reflected in higher square-roots of loss variance (sLV)—a parameter for the variability in the visual field.

At early stages, funduscopic examination reveals cupping of the optic nerve head (ONH) and loss of the neuroretinal rim and the retinal nerve fiber layer [5]. However, a significant inter-individual variability of the ONH morphology complicates both reliable identification of the relevant features (such as cup-disc ratio or neuroretinal rim area) in early glaucoma by the clinician as well as the classification based on quantitative measures of these features. Jonas et al. have proposed standardized clinical stages based on the progression of these features [5]. Even though advances in ocular imaging have revolutionized the reliable quantification of all aspects of optic nerve morphology, classification based on one single parameter is still not possible, and final diagnosis is based on clinical judgement [6]. Therefore, the diagnosis of pre-perimetric glaucoma can be associated with some uncertainty depending on the degree of morphological changes (glaucoma suspects).

Many functional techniques have been proposed for early detection of functional damage. Techniques based on psychophysics include short-wavelength automated perimetry [7], frequency doubling perimetry [8–10], flicker-defined-form perimetry [11, 12], rarebit perimetry [13, 14] and high-pass resolution perimetry [15, 16]. Electrophysiological techniques include PERG [17] and photopic negative response [18]. These techniques were also used to estimate the probability of the subject having glaucoma more reliably. Such techniques are commonly validated against clinical judgements performed by glaucoma experts, but this is not an ideal gold standard and the development of definite glaucomatous visual field defects over time is a more objective outcome.

The problem that remains when evaluating the diagnostic value of biomarkers in glaucoma is the lack of a good reference standard for early glaucoma, because optic nerve head morphology has large inter-individual variability [19] and because visual field defects cannot be reliably measured until a significant number of ganglion cells are lost [20]. Validation of diagnostic parameters against clinical judgement or available surrogate parameters may underestimate the quality of novel parameters that outperform these “gold standards,” and may yield incorrect results when prediction progression to blindness [21, 22].

Promising electrophysiological biomarkers for early functional detection of glaucoma are the pattern-ERG (PERG) and the blue-on-yellow VEP. For the PERG, a corneal or a skin electrode placed close to the globe records sum potentials generated by the retina as a response to the reversal of a checkerboard [17]. In this sum potential, responses from the outer retina are thought to be canceled out, because the time and space averaged retinal illuminance remains constant and because the outer retinal response to luminance increments and decrements are mirror images of each other. The remaining non-linear responses [23] are generated by the different mechanisms in the inner retina depending on check size [24]. Pathological PERG responses have been demonstrated in glaucoma [24–27], with response amplitude turning out to be a more useful parameter than implicit time [27]. Although the PERG may already be markedly decreased in ocular hypertension [25], it can be in the lower normal range even in advanced glaucoma [28], and the inter-individual variability is relatively high. Reversibility of changes in the PERG amplitudes has been demonstrated after lowering of IOP, suggesting that PERG detects injury in viable RGCs [29–31]. Importantly, Bach and coworkers have demonstrated in a prospective longitudinal study that the ratio of PERG amplitudes for small and for large checkerboards decrease before ocular hypertension converts to glaucoma and that the measurements four years before conversion had a good predictive power with an area-under-the-curve (AUC) of 0.75 in the receiver-operator characteristics (ROC) analysis [32, 33].

For pattern *visual-evoked potentials,* similar stimuli are used, but electrical responses of the occipital cortex are measured using electroencepholography, which are synchronized with the stimulus and averaged to cancel out other brain activity [34]. Here, further inter-individual variability is introduced through variations in the structure of the occipital cortex. Chromatic blue-yellow checkerboards (blue-yellowVEP: byVEP) have proven to be powerful in glaucoma diagnosis and implicit times of wave components were superior to the highly variable amplitudes [35, 36].

Survival analysis gives a theoretical framework for statistical analysis of the risk for an event – here the development of definite glaucomatous visual field defects – over time in cases of incomplete follow-up [37, 38]. These methods have already been used frequently in glaucoma research [39–42], but to our knowledge not for electrophysiological parameters.

The Erlangen Glaucoma Study (also Erlangen Glaucoma Registry) is a large prospective project that started in 1991 for evaluating morphological and functional parameters in diagnosing glaucoma. A considerable number of subjects participated in cross-sectional PERG and byVEP measurements [27, 35, 36, 43, 44].

In the present retrospective study, we used univariate and multivariate survival analyses to determine the value of PERG amplitude and byVEP peak time at baseline for predicting the risk of the development of visual field defects in glaucoma suspects, and to compare these parameters with perimetric sLV and ONH morphology.

## Methods

### Participants

The Erlangen Glaucoma Study is a longitudinal observational study including glaucoma suspects and patients with primary or secondary open angle glaucoma, whose age was between 18 and 65 years at baseline. Healthy volunteers were included mostly for cross-sectional analyses, but a small number were monitored longitudinally. Exclusion criteria were: Snellen visual acuity below 0.7, eye disease other than glaucoma, systemic diseases that potentially affect the retina or the optic nerve, especially diabetes mellitus, and myopic or hyperopic refractive error exceeding 8dpt.

**Fig. 1:**
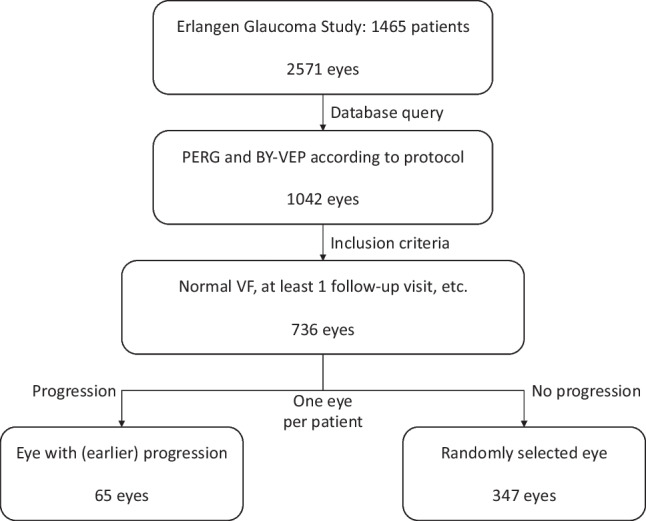
Flow diagram showing how data were selected for this retrospective analysis. Patients in the Erlangen Glaucoma Study participate in annual follow-up examinations including SAP and morphometric analysis of the optic nerve head

This retrospective analysis included patients (1) who had undergone PERG and BY-VEP measurements between 1991 and 2001, (2) who had normal visual fields at baseline, and (3) who had returned for at least one follow-up visit. Exclusion criterion for this analysis were X-chromosomal color vision defects as determined with the Nagel anomaloscope. Only one eye of each patient was included. If both eyes fulfilled the inclusion criteria, the eye with (earlier) progression was selected, or one eye was randomly selected if there was no progression in either eye (see Fig. 1).

The study was approved by the Ethics Committee of the medical faculty of the Friedrich-Alexander-University Erlangen-Nürnberg and followed the tenets of the Declaration of Helsinki for research involving human subjects. Written informed consent, including agreement for data collection, was obtained from all participants.

### Clinical examinations

Annual visits included a complete ophthalmological examination including slit lamp inspection, applanation tonometry, gonioscopy, funduscopy, standardized qualitative and quantitative analyses of color photographs of the optic nerve head, white-on-white standard automated perimetry (SAP). Scanning laser ophthalmoscopy was performed at baseline using the Heidelberg Retinal Tomograph (HRT) for objective measurement of the cup-disc-ratio.

Full threshold measurements (3 phases) were performed with the Octopus 900 perimeter (Haag-Streit, Schlieren, Switzerland) using the G1 pattern that covers the central field up to 30° eccentricity. The first three measurements were excluded from the analyses to mitigate learning effects. Visual field defects were defined as a loss of > 10 dB in one and > 5 dB in at least two adjacent location or as a loss > 10 dB in two adjacent locations.

Using color fundus photographs, ONH morphology was classified according to the criteria by Jonas and coworkers [5].

### Electrophysiological measurements

All measurements were performed unilaterally, while the other eye was covered with an eyepatch. For both the PERG and the byVEP, stimuli were created with an optical bench using a two-channel Maxwellian view system with mechanical mirrors and two monochromators. The subjects were examined with natural pupils while fixating a cross hair target in the middle of the pattern.

For recordings, the skin was cleaned with 96% Ethanol and then gently rubbed with skin preparation gel (Nuprep; Weaver and Company, Aurora, CO). The impedances between the electrodes were less than 10 kOhm. After 10,000 times amplification (model EMP88, notch filter at 50 Hz; 3 dB points at 0.5 and 70 Hz; Pölzl, Munich, Germany), biosignals were averaged in a personal computer. The signals were acquired at 500 Hz sampling frequency using an analog-to-digital converter (model ME26; Meilhaus Electronic GmBH, Puchheim, Germany) and a custom-written acquisition program. To check for reproducibility, all measurements were performed at least two times in each eye.

### Pattern-reversal electroretinogram (PERG)

For the PERG measurements, only one channel of the Maxwellian view system was used [27]. This system projected a vertical high-contrast black-and-white, square-wave grating alternating in counterphase at 7.8 Hz (15.6 reversals per second) with a mean retinal illuminance of 4263 photopic Td and a spatial frequency of 0.88 cpd.

Responses were recorded with a carbon glide electrode attached to the patient’s lower eyelid with reference (gold cup) electrodes at the ipsilateral cantus.

The amplified responses within one 256 ms sweep, the responses to four pattern-reversal were acquired. Using discrete Fourier analysis, the amplitudes and peak times of the second harmonic component of a total of 120 pattern reversal responses were averaged and evaluated.

### Blue-on-yellow visually evoked potential (BYVEP)

For 200 ms onset-500 ms offset VEP stimulation, one channel was used to project a high-contrast grating (0.88 cyc/deg) of blue light (460 nm, 3.3 × 10^2^ phot Td) superimposed on a homogeneous yellow adaptation light from the second channel (570 nm, 1.3 × 10^4^ phot Td). The grating was smeared of the complete stimulus by a quickly vibrating mirror during the offset phase of stimulation. As a result, the stimulus was spatially homogeneous during the offset phase. Furthermore, onset and offset phases had identical mean luminances and mean chromaticities [43].

VEP recordings were acquired using gold cup skin electrodes filled with electrode paste (TEN Conductive and Adhesive Paste, GE Medical Systems Information Technologies, Freiburg, Germany) were placed on the subject’s scalp (1 cm above the inion) and attached with a headband. A reference electrode was placed on the right, and a ground electrode on the left earlobe. All measurements were repeated three times (number of averaged sweeps 60–100 per measurement). Peak times and amplitudes of the onset responses were extracted from the responses included in the statistical evaluation.

### Statistical methods

Statistical analyses were performed using SPSS 28 (SPSS-Inc., Chicago, USA) or the R language for statistical computing. All amplitudes and peak times of both the PERG and the BY-VEP were age-corrected by performing a linear regression between response parameter and age for a control group of healthy volunteers [35, 36, 44–46].

Receiver-operating characteristic (ROC) curves and the optimum cut-points for dividing continuous independent variables into two categories (normal and abnormal) were obtained [47]. For the BY-VEP we used peak time as the outcome parameter, but for PERG we used amplitude based on our experience from earlier studies [27, 35].

Kaplan–Meier curves and the log-rank test were used to compare the rate of development of visual field defects between the Jonas classification (0: normal optic disc, I: concentrically enlarged cup, no focal notch, II: notching in the lower/upper temporal sector), and between normal and abnormal PERG amplitudes and BY-VEP peak times. For comparing the different modalities, a cox proportional hazard model was fitted to the data, including BY-VEP peak times, PERG amplitudes, and square-root of loss variance (corresponding to pattern standard deviation) of the SAP as parameters. The model was stratified for Jonas classification.

The level of significance was set at alpha = 0.05.

## Results

We identified 412 subjects from the Erlangen Glaucoma Study [47] (age at baseline 20–66 years) who fulfilled the inclusion criteria. The follow-up was between 6 month and 23 years (mean ± SD: 8.5 ± 7.3 years).

During this time, conversion to perimetric glaucoma was observed in at least one eye in 65 of 412 patients (15.8%) after 6 months to 20 years (mean ± SD: 9.7 ± 6.2 years). The descriptive characteristics are shown in Table 1.

**Table 1:** Descriptive characteristics at baseline, at the time of the final observation included in the survival analysis (time of conversion; in non-converters: final follow-up), and at the final follow-up in converters. The values refer to Mean (SD) or N (%) as appropriate

	Baseline		Conversion/Censoring		Final
	NP	P	NP	P	P
N	347	65			
Follow-up [years]			8.26 (7.4)	9.72 (6.2)	15.4 (6.5)
Female	155 (44.7%)	32 (49.2%)			
Age [years]*,**	45.4 (11.7)	51.6 (9.9)	53.7 (14.3)	61.4 (10.8)	67.1 (10.4)
BCVA: LogMAR*,**	-0.03 (0.05)	-0.01 (0.05)	0.02 (0.08)	0.06 (0.09)	0.10 (0.13)
Optic disc*,**					
0	277 (79.8%)	11 (16.9%)	261 (75.2%)	0	0
I	66 (19.0%)	37 (56.9%)	79 (22.8%)	36 (55.4%)	31 (47.7%)
II	4 (1.2%)	14 (21.5%)	7 (2.0%)	25 (38.5%)	26 (40.0%)
III	0	3 (4.6%)	0	4 (6.2%)	6 (9.2%)
IV	0	0	0	0	2 (3.2%)
Vertical CDR*,**	0.46 (0.24)	0.61 (0.17)	-	-	-
MD [dB] ^−^,**	-0.07 (1.20)	0.18 (1.17)	0.25 (1.40)	2.00 (1.7)	3.79 (3.5)
SLV*,**	1.54 (0.53)	1.75 (0.59)	1.68 (0.66)	3.08 (1.12)	4.36 (2.3)
Maximal therapy**					
No therapy	197 (56.8%)	17 (26.2%)	185 (53.3%)	-	0
Eyedrops only	121 (34.9%)	38 (58.5%)	111 (32.0%)	-	33 (50.8%)
LTP	26 (7.5%)	5 (7.7%)	36 (10.4%)	-	9 (13.8%)
Surgery	3 (0.9%)	5 (7.7%)	15 (4.3%)	-	23 (35.4%)
Pseudophakic	0	0	24 (6.9%)	4 (6.2%)	14 (21.5%)

NP: non-converting patients, P: converting patients; *) *p* < 0.05 between groups at baseline; **) *p* < 0.05 between groups at final visit

### Structure

**Fig. 2:**
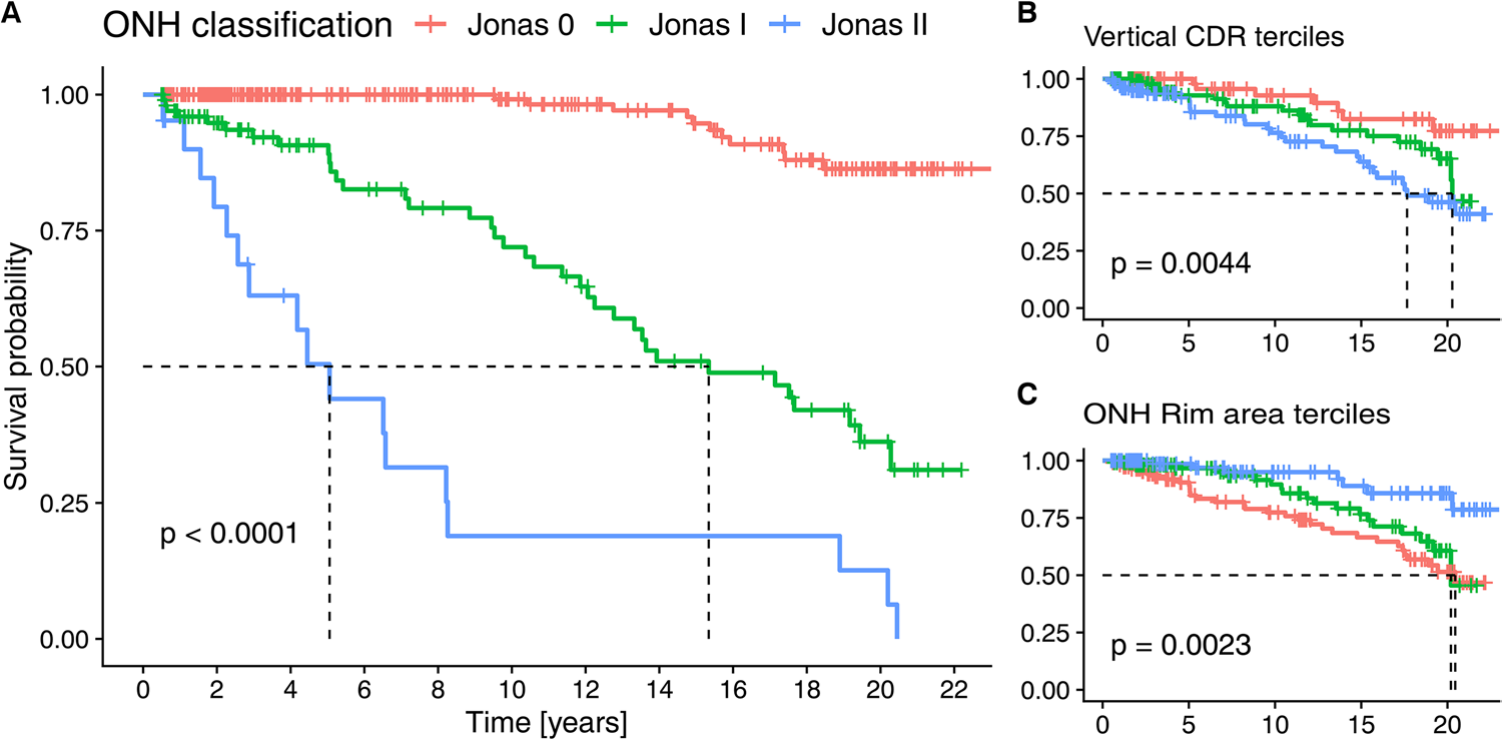
Kaplan–Meier curves for conversion to perimetric glaucoma. Panel **A** shows development of visual field defects depending on clinical classification of ONH morphology according to Jonas (0: normal ONH, I: concentrically enlarged cupping, II: focal notching a the supero- and/or inferotemporal rim). Panels **B** and **C** show the Kaplan–Meier curves for the morphologic parameters planimetric ONH rim area and vertical CDR (terciles, red: 1st, green 2nd, blue 3rd)

The time to conversion (TTC) depended strongly on the structure of the neuroretinal rim (see Fig. 2). Patients with more advanced neuroretinal rim loss progressed much earlier than those with normal neuroretinal rim (Schoenfeld test: *p* = 0.0208), even when under treatment according to standard clinical practices. The subgroup with a Jonas stage II had a median TTC of 5.1 years, while those with stage I had a median TTC of 15.3 years. In patients with stage 0, even the 25% conversion rate was > 22 years. The patients with different neuroretinal rim (NNR) classification differed significantly in cup-disc ratio (CDR), NNR area, but not in perimetric mean defect (see Table 2). However, the Jonas classification was superior to the planimetric neuroretinal rim area or vertical cup-disc-ratio (divided into terciles, see Fig. 2).

**Table 2:** Descriptive characteristics of the three Jonas classes, mean (SD) or *N* (%) as appropriate

	Jonas 0	Jonas I	Jonas II	*p*—value
N	288	103	21	
Age	44.7 (11.7)	49.8 (10.9)	52.8 (7.70)	< 0.001*
Female	127 (44.1%)	45 (43.7%)	15 (71.4%)	0.048*
BCVA: LogMAR	-0.03 (0.05)	-0.02 (0.05)	-0.01 (0.05)	0.037*
Spherical Equ. [D]	-0.88 (2.16)	-1.34 (2.51)	-0.35 (1.65)	0.085
PEX Syndrome	2 (0.69%)	0 (0.00%)	0 (0.00%)	1.000
Pigment Dispersion	11 (3.62%)	5 (4.85%)	0 (0.00%)	0.255
Baseline max. IOP [mmHg]	24.4 (6.70)	27.7 (6.56)	25.2 (6.45)	< 0.001*
C/D ratio	0.45 (0.25)	0.53 (0.20)	0.69 (0.09)	< 0.001*
NNR Area [mm^2^]	1.61 (0.39)	1.48 (0.46)	1.21 (0.31)	< 0.001*
Mean Defect [dB]	-0.08 (1.19)	-0.02 (1.18)	0.57 (1.15)	0.055

### Functional parameters

**Fig. 3:**
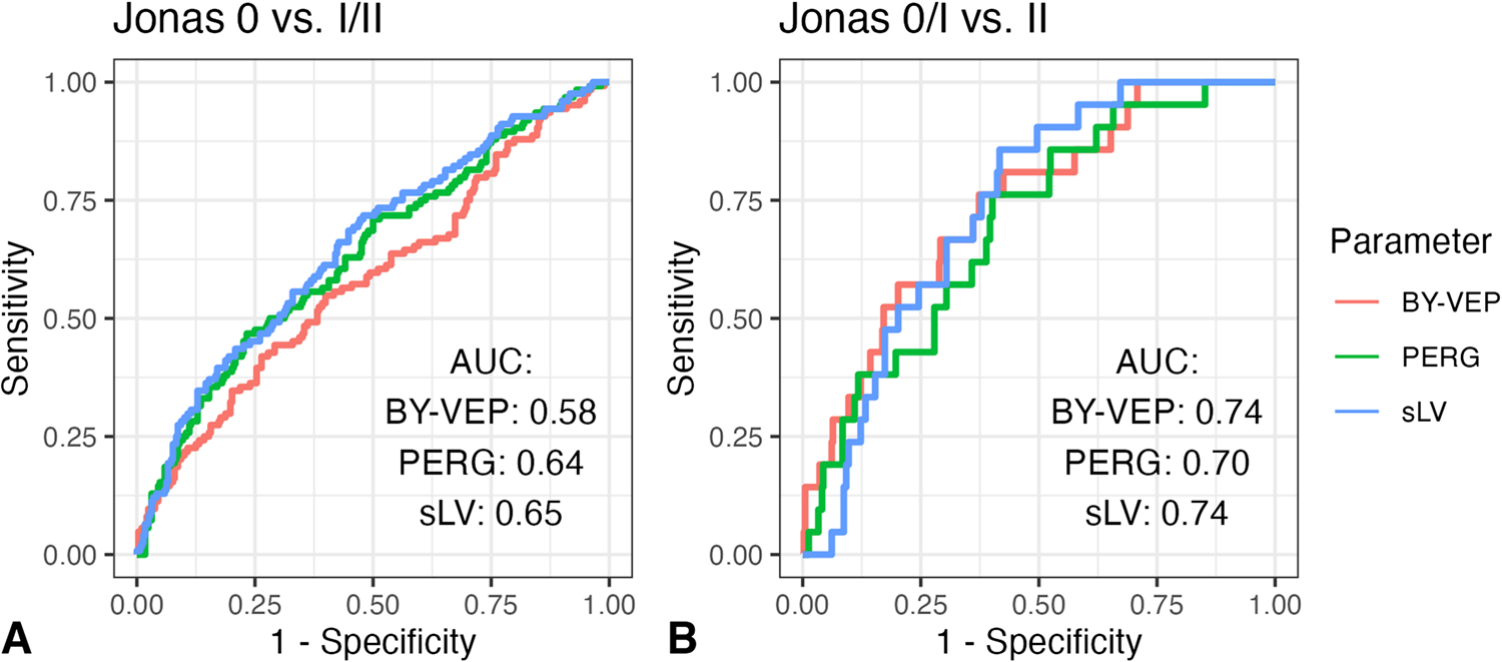
Ability of functional parameters to discrimination between Jonas stages. Panel A shows the ROC curve for normal optic nerve head (Jonas 0) vs. glaucomatous optic atrophy, while Panel B shows the ability to detect more advanced changes (Jonas 0/I vs. Jonas II)

The functional parameters BY-VEP latency, PERG amplitude and perimetry sLV were poorly correlated with the structural parameters neuroretinal rim area (PERG: *R* = 0.14, *p* = 0.01; BY-VEP: *R* = -0.17, *p* = 0.002; sLV: *R* = -0.14, *p* = 0.009) and vertical cup-disc-ratio (PERG: *R* = -0.26, *p* < 0.001; BY-VEP: *R* = 0.12, *p* = 0.02; sLV: *R* = 0.16, *p* = 0.003). Figure 3 shows that the ability of these parameters to discriminate between normal optic nerve head morphology (Jonas 0) and glaucomatous optic atrophy (Jonas I and II) is poor (area under the curve of 0.58 for BY-VEP, 0.64 for PERG and 0.65 for sLV), while the ability to discriminate between minimal changes (Jonas 0 and I) and more pronounced changes with temporal-superior and temporal-inferior notching is better (AUC of 0.74, 0.70, and 0.74, respectively). The cut-off derived from the Youden-index for Jonas 0 vs. Jonas I/II was nevertheless used for dichotomizing the results of the functional tests for the Kaplan–Meier analyses because there was a considerable rate of conversion also in the Jonas I group in Fig. 2.

### Blue-yellow-VEP peak times

**Fig. 4:**
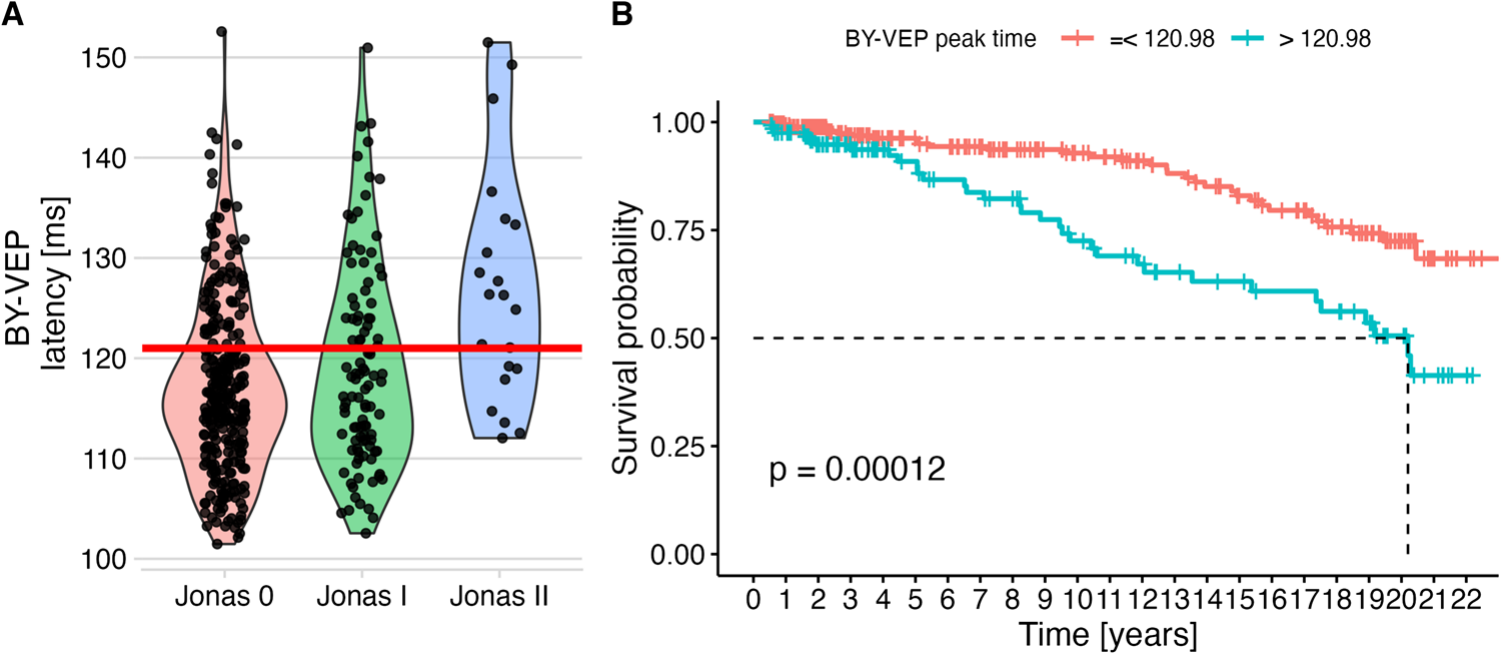
BY-VEP peak times and their predictive value. Panel A shows the observed values depending ONH classification. For survival analysis, a cut-off value (horizontal red line) was determined based on ROC analysis, optimizing discrimination between patients with normal ONH (Jonas 0) those with abnormal ONH (Jonas I, II, and III). Panel B shows that this parameter allowed prediction of conversion to perimetric glaucoma in this univariate analysis

The BY-VEP peak times were significantly different between groups (Jonas 0: 117 ± 8.6 ms, Jonas I: 119 ± 10.1 ms, Jonas 2: 127 ± 11.6 ms, *p* < 0.001). The cutoff determined by the Youden index for the ROC analysis discriminating normal ONH (Jonas 0) from abnormal ONHs (Jonas I and II) was 120.98 ms (Fig. 4A). Kaplan–Meier analyses are shown in Fig. 4B: the log-rank test was highly significant. In contrast, the BY-VEP amplitude did not yield a significant log rank test (*p* = 0.26) and this parameter was not used in the cox model.

**Fig. 5:**
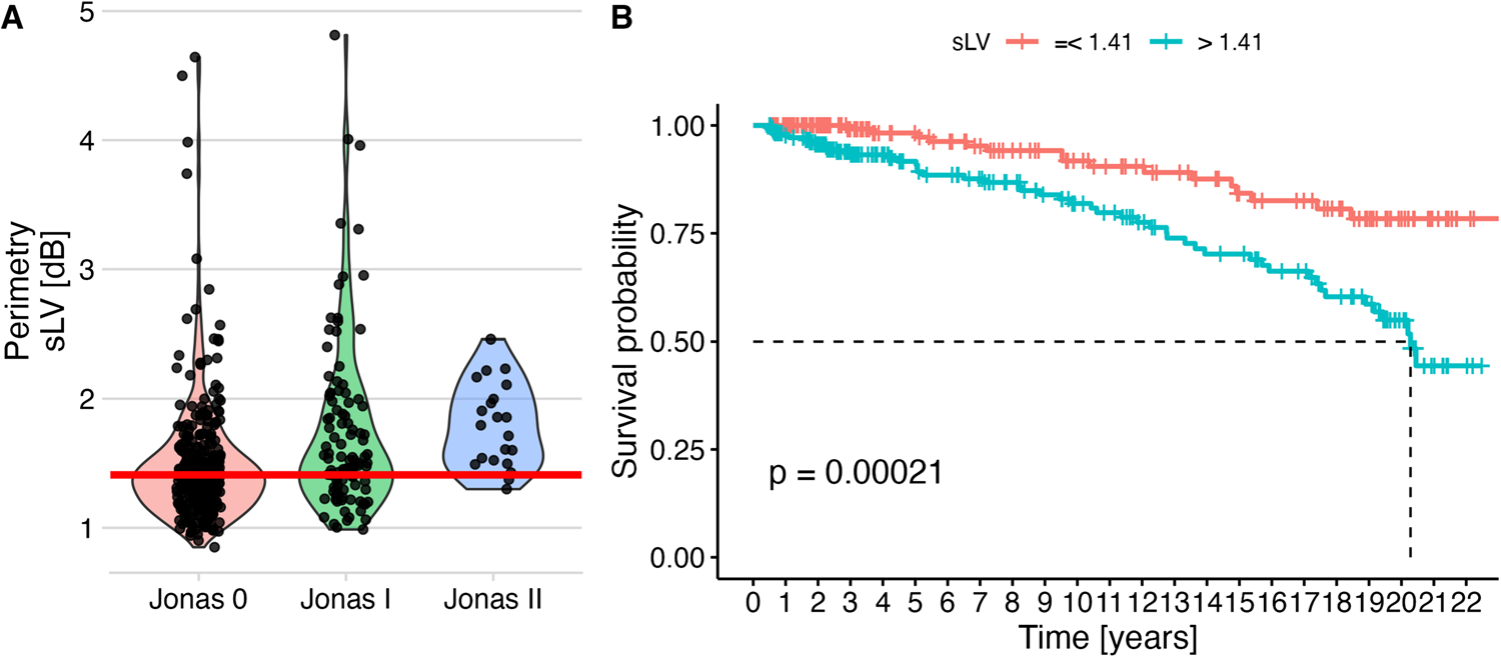
Perimetric square-roots of loss variance and their predictive value. As in Fig. 4, panel **A** shows the observed values depending ONH classification. For survival analysis, a cut-off value (horizontal red line) was determined based on ROC analysis, optimizing discrimination between patients with normal ONH (Jonas 0) those with abnormal ONH (Jonas I, II, and III). Panel B shows that this parameter allowed prediction of conversion to perimetric glaucoma in this univariate analysis

### Standard-automized perimetry: square-root of loss variance

SLV was not part of the definition of a perimetric defect and therefore could be used as an independent parameter for predicting conversion. Values ranged between 0.85 dB and 4.81 dB. There were significant differences between Jonas groups (Jonas 0: 1.49 ± 0.48 dB, Jonas I: 1.75 ± 0.67 dB, Jonas II: 1.79 ± 0.32 dB, *p* < 0.001). The optimal cut-off according to the Youden index was 1.41 dB (Fig. 5A), and this cutoff resulted in highly significant a log-rank test (Fig 5B).

### PERG amplitudes

**Fig. 6:**
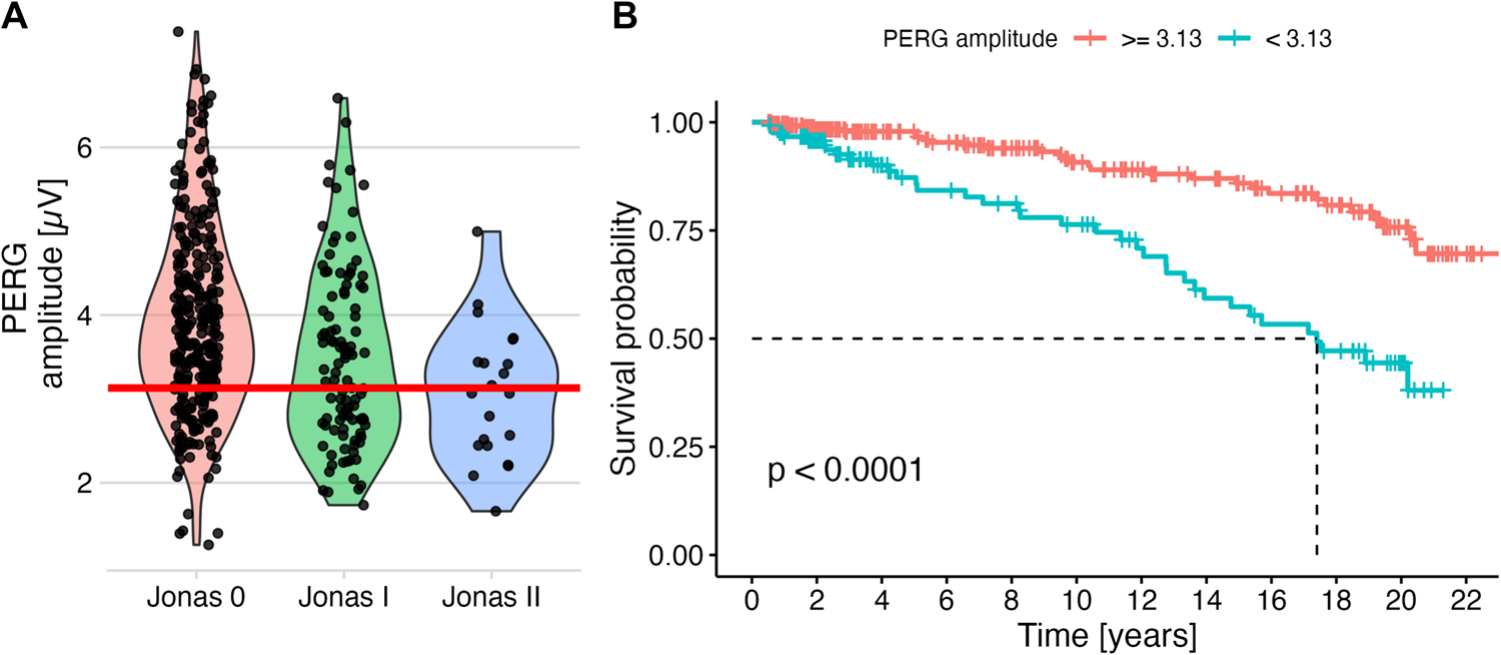
PERG amplitudes and their predictive value. As in the previous plots, panel **A** shows the observed values depending ONH classification. For survival analysis, a cut-off value (horizontal red line) was determined based on ROC analysis, optimizing discrimination between patients with normal ONH (Jonas 0) those with abnormal ONH (Jonas I, II, and III). Panel **B** shows that this parameter allowed prediction of conversion to perimetric glaucoma in this univariate analysis

PERG amplitudes ranged between 1.26 µV and 7.38 µV (3.78 ± 1.12 µV. They were also significantly different between Jonas stages (Jonas 0: 3.92 ± 1.13 µV, Jonas I: 3.49 ± 1.06 µV, Jonas II: 3.07 ± 0.80 µV, *p* < 0.001). A cutoff of 3.13 µV (Fig. 6A) resulted in a highly significant log-rank test (Kaplan–Meier analyses are shown in Fig. 6B). In contrast, the PERG peak time did not yield a significant log rank test (*p* = 0.18), and, therefore, this parameter was not used in the cox model.

### Multivariate analysis: Cox proportional hazards model

**Fig. 7:**
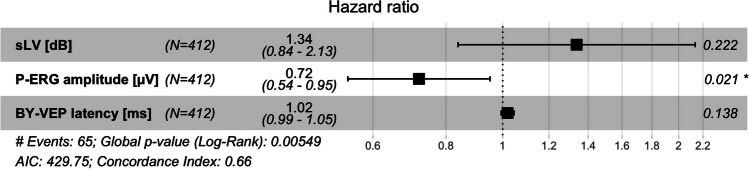
Forest plot of the cox proportional hazards model with stratification for NRR classification. Pattern-ERG amplitude remains an independent risk factor, with a reduction in the risk of conversion to perimetric glaucoma around 30% with each µV increase in amplitude

A multivariate analysis included the parameters above. Because survival analysis of the Jonas classification demonstrated that survival as a function of time was not proportional, it could not be included as a parameter. Instead, analysis was stratified for the three classes, which allows accounting for differences in the analysis of the other parameters, but no direct analysis in this model. Therefore, Jonas class is not included in the Forest plot in Fig. 7. Pattern-ERG amplitude remained an independent risk factor, with a reduction in the risk of conversion to perimetric glaucoma around 30% with each μV increase in amplitude.

## Discussion

We found that PERG amplitude was the only parameter that remained significantly associated with the risk for conversion to perimetric glaucoma in the multivariate analysis after stratification for ONH morphology. A decrease in baseline PERG amplitude of 1 µV corresponded to a ~ 30% increase in the risk of conversion. In contrast, the effects of BY-VEP peak time and perimetric SLV were significant only in the univariate analyses. Possibly, this is because PERG amplitude has the ability to demonstrate reversible dysfunction of RGCs [29, 30].

In our cohort, classification of ONH morphology according to Jonas was a strong predictor of the development of visual fields defects. The predictive value of ONH morphology has been demonstrated before for neuroretinal rim (NRR) area and peripapillary atrophy [40], but the predictive value of the Jonas classification seemed much more convincing in our study. Therefore, the Jonas classification might be a sufficient surrogate marker for the validation of glaucoma biomarkers in cross-sectional studies [21].

All three functional parameters were rather loosely correlated with ONH morphology. This is reflected in the poor correlation with both NRR area and vertical CDR, but also in the large overlap in the functional parameters between ONH stages according to Jonas. Our functional markers PERG amplitude and BY-VEP are dominated by macular responses. This might explain the poor correlation with *total* NNR area and vertical CDR ratio. Garway-Heath et al. have found a better correlation between steady state PERG and *temporal* NNR area (*R*^2^ = 0.2, *p* < 0.0001) [48]. The large overlap with increased latency or decreased amplitude corroborates earlier results that electrophysiological abnormalities are present in ocular hypertension (a considerable number of patients in the Jonas-0-group, but not all, have ocular hypertension) [49–51]. A more recent study has shown that an estimate of retinal ganglion cell count based on both perimetric mean defect and retinal nerve fiber layer thickness measured with OCT mediated the relationship between PERG and structural parameters [52].

The fact that morphological classification is much more easily available in clinical practice means that the electrophysiological markers only have a value if they yield *additional information* for predicting progression. However, this additional value can only be demonstrated in longitudinal studies. In our study, only PERG yielded additional information. This may indicate that PERG amplitudes detects changes independent of morphologically visible RGC loss. This is supported by the known discrepancies between PERG amplitude and morphological changes (decreased amplitudes in ocular hypertension and responses in the normal range in advanced glaucoma) [25, 28], and by the fact that PERG amplitudes have been shown to recover after IOP lowering or neuroprotective measures in glaucoma patients [29–31].

Altogether, the conversion rate in the cohort that we studied was low (median ~ 5 years in the high-risk patients with supero- and inferotemporal notching, ~ 15 years in patients with concentrically enlarged cup). Still, a lower PERG amplitude at baseline was associated with a higher risk for conversion over the course of many years. In another longitudinal study, Bach and coworkers found that PERG amplitude ratios reliably predicted conversion to glaucoma four years in advance (ROC: AUC of 0.75) [32, 33]. On the other hand, the authors reported that measurements more than 5 years before conversion did not have good predictive capability [51]. This is not in contrast with our findings, because ROC analysis requires a much closer relationship between predictor and outcome than cox proportional hazard models. The study by Bach and coworkers has several advantages over our study. It is a prospective, longitudinal study with regular PERG measurements and the PERG ratios seem to be better suited for predicting glaucoma than the amplitudes [33]. However, annual PERG measurements may not be feasible in many clinical settings and our study corroborates the value of PERG by showing that even one measurement may help to identify an increased risk many years in advance. Furthermore, survival analysis is superior for longitudinal analyses when patients are lost to follow-up [37, 38].

### Limitations

Modern tools like spectral domain OCT RNFL thickness measurements and retinal ganglion cell layer thickness were not available when baseline measurements were performed. We cannot rule out that these tools would have demonstrated more subtle structural changes that yield the same information as the PERG amplitudes.

We did not perform repeated PERG and BY-VEP measurements and we did not analyze treatment effects. Therefore, we were not able to identify patients where conversion was prevented by treatment.

PERG and BY-VEPs were measured with an optical bench and a Maxwellian view setup. This setup cannot be used in clinical practice, but recent studies using widely available computer screens support that more practicable devices also have a high validity.

Finally, we have performed multiple testing (8 logrank tests and one cox proportional hazards model) and not corrected for multiple testing. This was done because the logrank tests were exploratory analyses and the cox model was the single primary outcome. For example, the logrank test for the vertical cup disc ratio was statistically significant but the vertical cup disc ratio was not reported as an important result. The purpose of this test was merely to decide which morphological parameter has the best diagnostic value and should be used for stratification of the Cox model. Also, we do not propose to use BY-VEP as a biomarker despite a statistically significant logrank test. Therefore, multiple testing does not increase the risk of a type I error impacting the conclusion of our study.

### Implications for clinicians

The value of PERG and BY-VEP for detection functional defects in glaucoma have been demonstrated decades ago. They are not widely used today for two reasons: 1) electrophysiological measurements are difficult to obtain and to interpret outside specialized labs, and 2) compared with SAP, the structure–function relationship is more complicated and less well studied.

However, our studies show that electrophysiological parameters like PERG amplitude could potentially increase the value of multifactorial models for predicting the risk of conversion to perimetric glaucoma in patients at risk. Technical advances will facilitate these measurements in glaucoma clinics in the future.

Further research is needed to investigate whether improvement of PERG amplitude under neuroprotective therapy is associated with a decrease in the risk of conversion.

## Conclusions

Lower PERG amplitudes were associated with increased risk of development of visual field defects, independently of morphological parameters. Possibly, this is because PERG allows demonstration of dysfunction in viable ganglion cells.
